# Application of Chaotic Laws to Improve Haplotype Assembly Using Chaos Game Representation

**DOI:** 10.1038/s41598-019-46844-y

**Published:** 2019-07-17

**Authors:** Mohammad Hossein Olyaee, Alireza Khanteymoori, Khosrow Khalifeh

**Affiliations:** 1 Department of Computer Engineering, University of Zanjan, Zanjan, Iran; 2 Department of Biology, Faculty of Sciences, University of Zanjan, Zanjan, Iran

**Keywords:** Computational models, Genome assembly algorithms

## Abstract

Sequence data are deposited in the form of unphased genotypes and it is not possible to directly identify the location of a particular allele on a specific parental chromosome or haplotype. This study employed nonlinear time series modeling approaches to analyze the haplotype sequences obtained from the NGS sequencing method. To evaluate the chaotic behavior of haplotypes, we analyzed their whole sequences, as well as several subsequences from distinct haplotypes, in terms of the SNP distribution on their chromosomes. This analysis utilized chaos game representation (CGR) followed by the application of two different scaling methods. It was found that chaotic behavior clearly exists in most haplotype subsequences. For testing the applicability of the proposed model, the present research determined the alleles in gap positions and positions with low coverage by using chromosome subsequences in which 10% of each subsequence’s alleles are replaced by gaps. After conversion of the subsequences’ CGR into the coordinate series, a Local Projection (LP) method predicted the measure of ambiguous positions in the coordinate series. It was discovered that the average reconstruction rate for all input data is more than 97%, demonstrating that applying this knowledge can effectively improve the reconstruction rate of given haplotypes.

## Introduction

Deposited in bioinformatics databases is a wide range of available sequence data obtained from different high throughput sequencing tools. This wealth of data, when accompanied by advances in computational methods, has revolutionized the study of genome variation under the new emerging field of systems biology.

More than 99% of human genome is identical among individuals as well as different ethnic groups. In other words, less than 1% of genetic differences are responsible for all of the observed variations among people all over the world^1^.

Therefore, specifying these differences in genetic material and evaluating the distribution on the DNA sequences of different human populations may have important implications in solving various problems in biology and medicine. In line with this assumption, two leading projects, the international haplotype map (Hapmap)^2^ and 1,000 genomes^3^, have been pursued to characterize common patterns of human genetic variations.

Single nucleotide polymorphisms (SNPs) are the most common types of genetic variations in the human genome. SNP refers to the occurrence of different single nucleotides at specific positions in the human genome, which resulted from mutations followed by natural selection during the evolutionary time scale. The possible nucleotides define alleles for that position^4^. A SNP sequence along each chromosome is known as a haplotype. Both SNPs and haplotypes provide valuable information for assessing genetic variations in a systematic manner. Different research fields, such as disease susceptibility, drug design, and genome-wide association studies (GWASs)^5^, can greatly benefit from this data.

The distribution of SNPs across genome elements has been investigated by a multitude of studies. These have illustrated that SNPs tend to be clustered across the genome elements in a deterministic manner in which the position of the each mutation is usually affected by its neighbors and the sequences of SNPs are often highly correlated with each other^6–8^. Based on this finding, several studies have proposed models to describe how SNPs clustered along the genome sequence lead to the construction of haplotypes^9–11^. In order to identify genes involved in genetic diseases, massive amounts of SNP and haplotype data were utilized by GWASs to detect highly statistically significant correlations between SNPs on the genetic materials and various numbers of phenotypes^12^. (https://ghr.nlm.nih.gov/primer/genomicresearch/gwastudies). These are essential for the prediction, diagnosis, prevention or medical therapy of diseases by contextualizing reference big data and provide the basic elements of modern personalized medicine^13–16^. Accordingly, identifying haplotypes, particularly when input fragments contain large sections of gaps without enough coverage is critical. In order to handle reading errors, low coverage, and large number of input fragment gaps, several fragment assembly algorithms have been proposed to reconstruct the haplotypes from fragments of homologous human chromosomes from a single individual^17–24^. The identification of correlation between SNPs is the key challenge of recognizing haplotype sequences. Chaos theory provides a powerful tool for discriminating between random and deterministic processes if a suitable phase space embedding can be found. Indeed, several studies have shown that the underlying information structure can be revealed by chaos theory without the reliance on the respective equations of systems dynamics^25^. Based on this assumption, Chaos theory has been increasingly applied in Life sciences for understanding the complexity of biological systems^26^. For example, many attempts have been made to explain the chaotic behavior of biological sequences^27–32^. Moreover, chaotic view point has been applied to evaluate biological signals such as electroencephalogram (EEG) signals^33–35^.

Mapping protein sequences in 2D space with chaos game representation (CGR) has shown that the structural classes of proteins can be distinguished by comparing their chaotic behavior^36^. CGR is an iterative mapping algorithm which was initially developed by Jeffrey^37^ for visualizing genomic sequences as chaotic systems^38^. This method can transform an input one dimensional biological sequence into an intuitive two dimensional picture^39^.

This study utilized nonlinear chaotic analysis with a surrogate data test and multi-fractal analysis to determine whether haplotypes can be detected as non-random SNP sequences. Also NA12878 dataset was used in binary form containing haplotype sequences of all human chromosomes.

Since SNPs defining haplotypes are highly correlated with each other, several subsequences are extracted from each haplotype sequence and each haplotype is locally evaluated. After the CGR method transformed each subsequence to a line, the corresponding coordinate series was extracted and its chaotic behavior was evaluated by a surrogate test. For more detailed assessment, a multi-fractal spectrum of the sequences was also computed. The resulting data confirmed that haplotype sequences of representative chromosomes originate from a non-stochastic process involving the neighbor effect of its constituents.

In order to test the ability of the proposed model to accurately manipulate haplotype sequences, single individual haplotype (SIH) reconstruction as a complicated task in computational biology was taken into account and the knowledge of chaotic behavior was utilized to improve the rate of haplotype reconstruction. The main concern of SIH is the reconstruction of haplotypes from several input fragments originating from a given sequencing method. As mentioned earlier, sequencing errors and missing information (gaps) are the main challenges in dealing with this problem. Existing methods suffer from huge numbers of gaps as these lead to positions with low coverage and thus low confidence in attempts to identify alleles in such areas^4^. Here, the current work mapped each haplotype by CGR and extracted a coordinate series in the same way as previously described. The Local Projection (LP) method then locally estimated the trajectory in the neighborhood of each ambiguous point and, finally, each ambiguous point was determined by a projection to the resultant curve. The experimental results revealed that utilizing the knowledge of chaotic behavior can help improve the reconstruction rate and also play a complementary role in the existing methods.

## Materials and Methods

In order to provide a comprehensive analysis of biological sequences, the current study applied a five-step rule, as proposed by Chou^40^, in the following order: (a) provide a valid dataset to evaluate the hypothesis; (b) express biological sequences with appropriate mathematical notations while preserving all of their hidden information; (c) explain the proposed method exactly; (d) evaluate the final results; and (e) provide the source code of implementations.

### Materials

The current work’s dataset included the HapMap NA12878 Whole-Genome Sequence (WGS) sample for a European (i.e., CEU) female individual, also known as HG001, which is a well-known reference genome dataset containing haplotype sequences of all human chromosomes^41,42^. The reference haplotypes were the trio-phased variant calls from the GATK resource bundle^43^. They were produced by a fosmid-based technology from the HapMap sample NA12878 and filtered in 1,252,769 positions that were also covered by fragments of the NA12878 dataset.

### Chaos game representation

CGR is a well-known algorithm which iteratively maps an input sequence into 2D space. This mapping leads to visualization of the input sequence in a picture. Furthermore, this procedure can reveal the hidden patterns of subsequences^28^.

For sequences with four alphabets, such as DNA, the final picture takes on a square format. Each vertex equals one nucleotide, i.e. A, T, C, and G. The sequence is mapped in the area of the square with a unit length such that each nucleotide base is plotted as a point. The first point is plotted at the center of the square. Next, the first base is placed halfway between the center of the square and the vertex which corresponds to the first base. As seen in the following formula, the coordinate of the i^th^ base (b_i_) is placed halfway between the (i − 1)^th^ point and the respective vertex (v_i_).

1 $${b}_{i}=0.5\times ({b}_{i-1}+{v}_{i})a$$

The plot is known as the CGR of the input sequence.

### Analysis of nonlinear time series

Suppose *X*_*t*_ is a scalar time series where *t* = *1*, 2*,…, N*. If this time series is observed from a deterministic phenomenon perspective, it can be projected into a low dimensional state space called phase space. If ${Y}_{t}=\{{x}_{t},{x}_{t+\tau },\ldots ,{x}_{t+(m-1)\tau }\}$ is *X*_*t*_ in the phase space, then the phase space can be reconstructed according to Takens’ embedding theorem^44^. For this purpose, parameters $\tau$ and *m*, as the time delay and embedding dimension respectively, should be determined. Dimension *m* completely demonstrates the object and its topological features. There are several approaches, such as Average Mutual Information (AMI)^45^ and False Nearest Neighbor (FNN)^46^, which heuristically estimate phase space parameters based on the available data.

### Lyapunov exponent

Sensitive dependence on initial conditions is one of the main properties of chaotic systems. For an m dimensional chaotic system, the Lyapunov exponent (*λ*) is a spectrum containing m real numbers which quantifies sensitivity to initial conditions. It should be noted that the sign of its largest measure is positive for chaotic systems and its quantity indicates the extent of the chaotic system’s predictability.

Suppose *Y*(0) and ${Y}_{{\epsilon }}(0)$ are two initial neighbor points in phase space, in which$\Vert Y(0)-{Y}_{{\epsilon }}(0)\Vert ={\epsilon }$. By the evolution of time, the points are separated and the average of this separation, equaling *λ*_*max*_, is obtained according to the following equation:

2 $${\lambda }_{max}={\mathrm{lim}}_{t\to \infty }\mathop{\mathrm{lim}}\limits_{{\epsilon }\to 0}\frac{1}{t}\,\mathrm{ln}(\frac{\Vert Y(t)-{Y}_{{\epsilon }}(t)\Vert }{{\epsilon }})$$

Although calculating *λ* from experimental data is a difficult task, several methods have been proposed to determine the largest Lyapunov exponent^47–50^. In this study, the Eckmann’s method^51^ was chosen because it is one of the most practical approaches for determining the Lyapunov exponent from the experimental data^52^. The first step involves mapping *X*_*t*_ as a scalar time series to ${Y}_{t}=\{{x}_{t},{x}_{t+\tau },\ldots ,{x}_{t+(m-1)\tau }\}$ by reconstructing the phase space. Suppose *Y*_*j*_ and ${Y}_{j+{\tau }_{2}}$ are two points in the phase space such that $a{Y}_{j+{\tau }_{2}}$ is the evolution of *Y*_*j*_ which has been provided by a rule or map as below:

3 $$F({Y}_{j})={Y}_{j+{\tau }_{2}}$$

In the above relation, *τ*_2_ is the iteration step which can be selected independently from *τ*. In the next step, for each point *Y*_*j*_, all of its neighbors is found. Suppose ${Y}_{j}^{r}$ is the r^th^ nearest neighbor of *Y*_*j*_, calculating the Lyapunov exponent involves determining $\underline{D}\,F({Y}_{j})$ which maps all neighborhoods of vectors ${Y}_{j}^{r}-{Y}_{j}$ to ${Y}_{j+{\tau }_{2}}^{r}-{Y}_{j+{\tau }_{2}}$. It should be noted that $\underline{D}\,F({Y}_{j})$ is the *m* × *m* Jacobian matrix of *F* at *Y*_*j*_.

The Lyapunov exponent is obtained by calculating the eigenvalues of the matrix ($\underline{D}\,{F}^{K})^{\prime} \underline{D}\,{F}^{K}$ where $\underline{D}\,{F}^{K}$ is computed as below:

4 $$\underline{D}\,{F}^{K}=\underline{D}\,F(K).\,\underline{D}\,F(K-1)\ldots \underline{D}\,F(1)$$

where *K* is an arbitrary integer of evaluation points, and $\underline{D}\,F(K)=\underline{D}\,F({Y}_{K})$.

### Correlation dimension method

Suppose X is a chaotic time series whose attractor has been reconstructed in phase space. The correlation dimension method is one of the most fundamental approaches for studying chaotic time series, by which its measure describes the complexity of the attractor^53^. The correlation dimension can be expressed by Equation (5):

5 $$C(r)=\frac{2}{(N)(N-1)}{\sum }_{i,j=1}^{N}H(r-\Vert {Y}_{i}-{Y}_{j}\Vert )$$

where *N* is the number of m-dimensional points on the reconstructed space, *Y*_*i*_ is the delay vector, *r* is a neighborhood, and *H* is the Heaviside step function. *C(r)* is computed for a range of neighborhood sizes *r* and a range of embedding dimensions *m*. The next step plots the slopes of *C(r)* against *r* on a log-linear plot. For each embedding dimension, there may be a specific curve. If these curves saturate on a common plateau, their y-value is a measure of the correlation dimension. The following describes the relationship between *r* and *C(r)*:

6 $$C(r)\propto \alpha {r}^{{D}_{2}}$$

where *α* is a constant value and *D*_2_ is the correlation dimension given by Equation (7):

7 $${D}_{2}=\mathop{\mathrm{lim}}\limits_{r\to 0}\frac{\mathrm{log}\,C(r)}{\mathrm{log}\,r}$$

As seen in the above formula, *D*_*2*_ is estimated based on the linear region, which is found between the depopulated and saturated regions. It should be noted that the depopulated region refers to the area of the plot with no pairs of points. The saturated region includes a large value of *r* where *C(r)* reaches a constant value.

It should be emphasized that the correlation dimension is suitable for situations in which the chaotic behavior of a given system is known. In other words, the correlation dimension is unable to distinguish between the stochastic and deterministic processes.

### Surrogate data test

Surrogate data test is a Monte Carlo-based algorithm which can detect the chaotic behavior of an existing time series. This test supposes that the given time series is random and is provided by a stochastic process. Then, an arbitrary amount of surrogate data is generated. These data are random but preserve the statistical properties of the original data. The test starts with the hypothesis that the original time series is random. Next, a method for nonlinear time series analysis is chosen, such as that of extracting a correlation dimension, and this measure is computed for the original and surrogate time series. If the results for the original time series are completely different with those for the surrogate time series, then it can be concluded that the hypothesis is not true. In other words, the original time series is related to a deterministic process. It is worth mentioning that the generated surrogate data cover most of the subset of the stochastic process class.

There are several ways to generate surrogate data, the most important of which consists of the following steps. First, Fourier transform converts the original time series to the frequency domain. Then, each element is changed by multiplication to a random phase with a unit magnitude. The resulting data are transformed back by inverse Fourier transform and, finally, randomized data with the same power, known as surrogate data, are generated.

### Multi-fractal analysis

Multi-fractal refers to elements composed of several simple fractal objects. Fractal dimension cannot describe these objects’ dynamic behavior. Instead, a continuous spectrum, namely the generalized fractal dimension, was developed^54^. When the attractor of a given time series is plotted in a phase space, this time series reveals chaotic behavior when the attractor is fractal or multi-fractal. Accordingly, multi-fractal analysis, as well as the surrogate data test, can help reveal the chaotic features of a given object. Up until now, several approaches have been proposed for implementing multi-fractal analysis. Fixed size box-counting is one of the most popular methods employed for solving various problems. As expressed in the following relationship, the surface of a given object is covered by several identical size *ε* boxes. *μ* is an arbitrary function which calculates the density of points (B) for each of the boxes. The partition sum of all non-empty boxes can then be calculated according to Equation (8):

8 $$\begin{array}{ll}{Z}_{\varepsilon }(q)={\sum }_{\mu (B)\ne }{[\mu (B)]}^{q} & q\in R\end{array}$$

In the above relationship, *q* can assume any real value for discriminating the sparse from the dense regions. Equation (9) calculates the mass exponent:

9 $$\tau (q)={\mathrm{lim}}_{\varepsilon \to 0}\frac{\mathrm{ln}\,{Z}_{\varepsilon }(q)}{\mathrm{ln}\,\varepsilon }$$

Finally, the generalized fractal dimensions are defined by the following relationships:

10 $${D}_{q}=\frac{\tau (q)}{(q-1)},\,{\rm{for}}\,{q}\ne 1$$

11 $${D}_{q}={\mathrm{lim}}_{\varepsilon \to 0}\frac{\sum \mu (B)\mathrm{ln}\,\mu (B)}{\mathrm{ln}\,\varepsilon }\,,{\rm{for}}\,{\rm{q}}=1$$

*f*(*α*) spectrum is used to evaluate the multi-fractal behavior of the data. Equation (12) expresses this measure:

12 $$f(\alpha )=q\alpha (q)-\tau (q)$$

Here, *α*(*q*) is the Lipschitz-Holder exponent which determines the singularities of a measure. This measure is related to *τ(q)* and is given by the following relationship:

13 $$\alpha (q)=\frac{d}{dq}\tau (q)$$

It should be noted that *f*(*α*) can determine the strength of multi-fractality, such that a narrower spectrum demonstrates weak multi-fractal behavior and a broader spectrum indicates stronger multi-fractality behavior.

## Results and Discussions

### Extracting subsequences

The analysis was performed on the full length sequences of distinctive haplotypes as well as the subsequences of haplotypes, as described below. Since the overall results of the full length analysis indicated that these sequences did not exhibit chaotic behavior, a detailed analysis of their subsequences is provided here.

**Figure 1:**
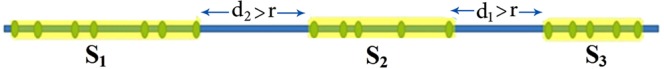
*S*_1_, *S*_2_, and *S*_3_ are three subsequences whose SNP distances are less than predefined r.

As shown in Fig. 1, a number of SNPs, whose distances were less than predefined threshold r, constructed subsequence S_i_. Since the extracted subsequences should have had the minimum data length required for chaos analysis, the present study assumed r equals 30,000 and selected subsequences whose lengths were greater than Thr (800) for further analysis.

By applying these cut-offs, different numbers of subsequences were extracted for each chromosome. Table 1 presents the total number of subsequences and those with lengths greater than the threshold value.

**Table 1:** Subsequence information extracted from all chromosomes.

Chromosome	#All Subsequences	#Subsequences with a Length >*Thr*
1	874	38
2	914	41
3	697	39
4	751	39
5	594	40
6	567	35
7	577	35
8	552	34
9	418	23
10	443	25
11	474	29
12	464	30
13	304	22
14	336	17
15	346	10
16	271	16
17	346	11
18	245	17
19	177	7
20	198	10
21	111	5
22	101	5

### Chaos game representation of haplotype sequences

**Figure 2:**
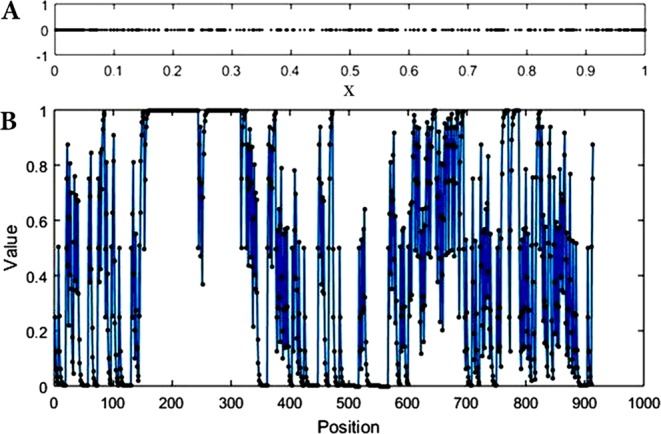
Binary CGR of the first extracted subsequence (**A**) and its extracted coordinate series (**B**) from the first subsequence of Chromosome 1. Since the input sequences consist of two alphabets, the obtained pictures resemble dotted lines.

CGR is an iterative mapping algorithm which can provide a visualize form for a biological sequence. Detailed examination of the obtained picture can reveal the chaotic behavior of a system in terms of the local patterns of the sequence^38^. In order to quantitatively assess the output of CGR, a coordinate series is extracted containing all positions of the CGR picture. A typical CGR picture and its corresponding coordinate series for the first subsequence of Chromosome 1’s haplotype are shown in Fig. 2’sA,B, respectively. It is worth mentioning that individual values of input sequences correspond to unique points in the CGR picture and vice versa.

Since all information is preserved in the CGR plot, as well as in its respective coordinate series, the resulting data of the coordinate series assessment can be attributed to its related CGR picture.

### Surrogate test

In order to examine the nonlinear properties of the original input data, the statistical surrogate data testing method was applied on individual coordinate series. The following procedure prepared the surrogate data. For each coordinate series, the embedding dimension and time delay were first determined and 10 surrogate coordinate series were then generated according to the method reviewed earlier. After this, the correlation dimension was computed for the original and its related surrogate coordinate series.

Table 2 presents the typical results of the surrogate test, including the correlation dimension values for the original coordinate series along with the minimum and maximum surrogates for all Chromosome 1 haplotype subsequences. The last column contains the values of the largest Lyapunov exponent (LLE) of each subsequence.

**Table 2:** Results of the surrogate test and LLE measures for the extracted subsequences of Chromosome 1’s haplotype.

	Original	Surrogate Min	Surrogate Max	LLE
S_1_	0.36627	0.39204	0.58594	0.33360
S_2_	0.13629	0.04635	0.05898	0.13629
S_3_	0.43052	0.71659	0.51951	0.34368
S_4_	0.26051	0.27251	0.54544	−0.05953
S_5_	0.35052	0.40979	0.57804	0.64071
S_6_	0.10796	0.10934	0.16225	0.26067
S_7_	0.25108	0.29168	0.38618	0.69047
S_8_	0.23778	0.25782	0.47653	0.24444
S_9_	0.35097	0.35113	0.62711	0.03415
S_10_	0.27321	0.26391	0.55738	0.47606
S_11_	0.31865	0.36589	1.00820	−0.05989
S_12_	0.10715	0.12004	0.15777	0.83138
S_13_	0.39225	0.45739	0.67238	0.30744
S_14_	0.47752	0.58567	0.75620	0.29638
S_15_	0.43977	0.49288	0.81505	0.03177
S_16_	0.18912	0.19767	0.30535	−0.03274
S_17_	0.53972	0.60917	0.95226	−0.10400
S_18_	0.27841	0.28615	0.42475	0.35364
S_19_	0.45505	0.50642	0.65790	0.33360
S_20_	0.47605	0.55843	0.90008	−0.10132
S_21_	0.18262	0.20794	0.39612	0.01682
S_22_	0.11989	0.13120	0.19777	0.83163
S_23_	0.10483	0.10412	0.13882	0.37704
S_24_	0.54638	0.29920	0.54770	1.13682
S_25_	0.07839	0.08140	0.12628	0.97159
S_26_	0.09959	0.09959	0.13728	0.60269
S_27_	0.13594	0.13708	0.25053	0.86333
S_28_	0.15408	0.16693	0.25944	0.76313
S_29_	0.43850	0.5150	0.83745	0.13629
S_30_	0.13553	0.13878	0.30403	0.18735
S_31_	0.09739	0.09993	0.18582	−0.03322
S_32_	0.14369	0.15750	0.22236	0.53379
S_33_	0.24935	0.28596	0.64447	0.43406
S_34_	0.16918	0.18759	0.25632	0.60329
S_35_	0.44667	0.47550	0.71674	0.35169
S_36_	0.30748	0.31783	0.72264	0.13042
S_37_	0.07777	0.07777	0.10733	−0.02983
S_38_	0.31624	0.39668	0.93261	0.34368

**Figure 3:**
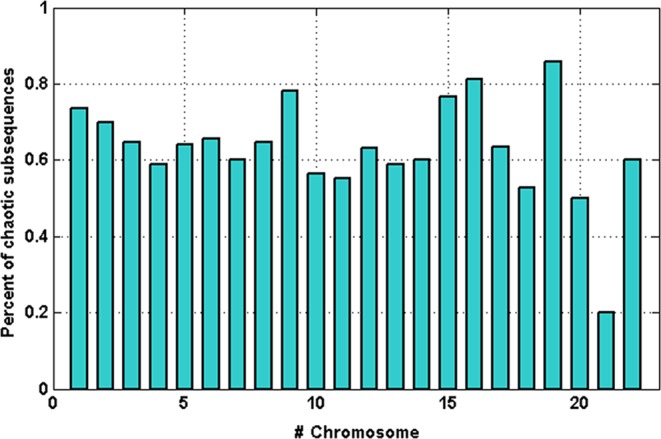
Percent of chaotic subsequences found throughout all chromosome haplotypes.

According to Table 2’s data, the null hypothesis of the surrogate test was rejected for most of the subsequences and the sensitivity for the initial condition was confirmed for subsequences containing positive LLE. Thus, Table 2’s data demonstrates that 74% of Chromosome 1’s haplotype subsequences exhibited chaotic features. The surrogate test and LLE computation were also carried out for the other chromosome haplotypes. As shown in Fig. 3, some percentages of subsequences in other haplotypes involved chaotic features. The resulting data indicate that most of the extracted subsequences exhibited chaotic behavior. Since mapping from sequences to coordinate series preserves all the information, the obtained results indicate that these subsequences originated from deterministic behavior.

### Multi-fractal analysis

**Figure 4:**
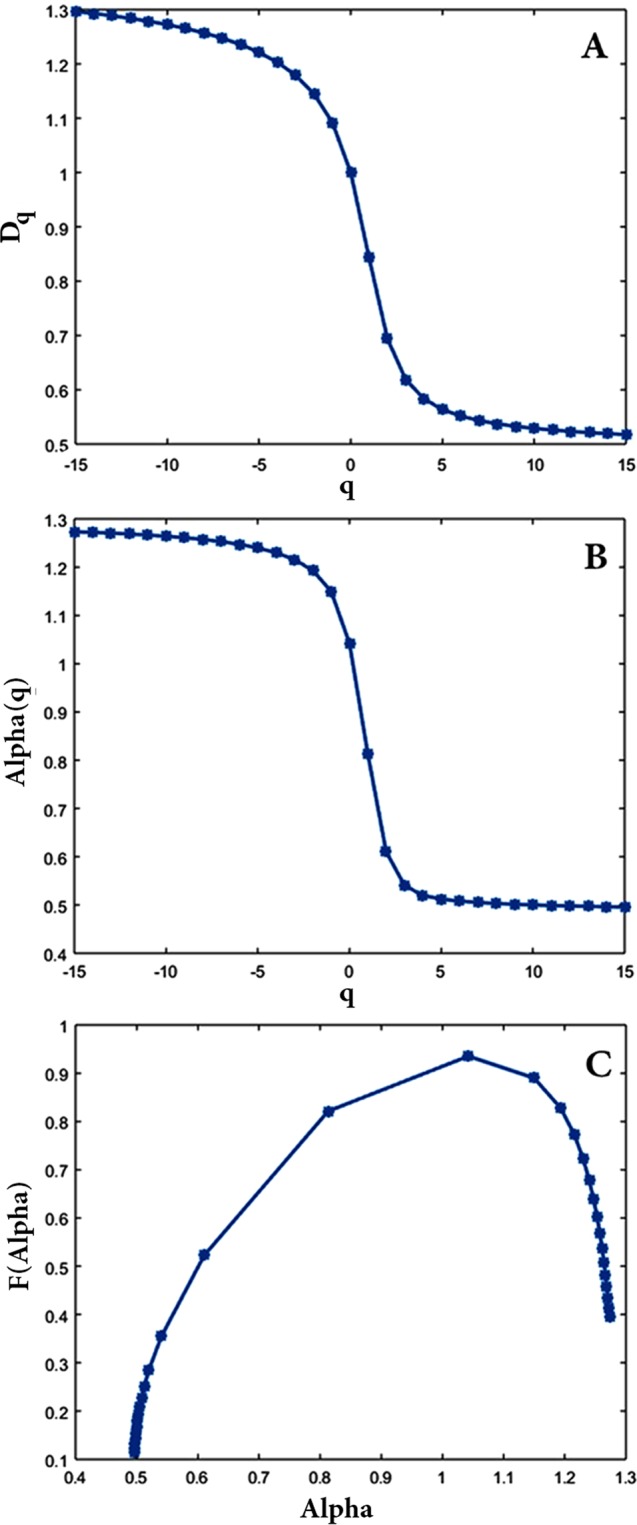
Three curves related to the first subsequence of Chromosome 1’s haplotype. (**A**) represents D_q_, (**B**) is related to spectra *α*, and (**C**) represents *f*(*α*).

To confirm the above results, whose findings observe chaotic behavior in coordinate series extracted from subsequences, the present study applied multi-fractal analysis for CGRs. Multi-fractal analysis is another method for examining chaotic behavior. For investigating the scaling behavior of the CGR picture, CGRs were covered by several size *ε* boxes. In line with this assumption, if ε was equal to $\frac{1}{8}$, for instance, the CGR image was covered by eight boxes. Here, the input sequences had two alphabets and so the resulting CGR was a dotted line. With this method of analysis and according to the density of points in each box, the multi-fractal parameters, including *D*_*q*_, *α*, and *f*(*α*), were calculated. Figure 4 provides the results of this calculation for the first subsequence of Chromosome 1’s haplotype.

**Figure 5:**
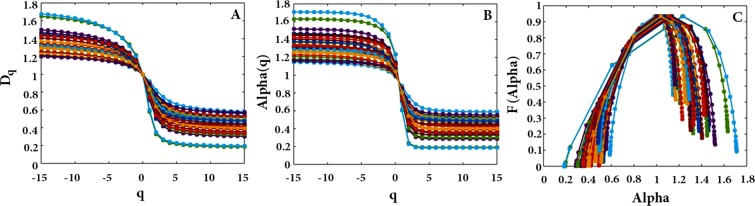
Multi-fractal curves of all subsequences related to Chromosome 1’s haplotype (**A**) dimension spectra, (**B**) specturm *α*, and (**C**) *f*(*α*). All of the selected subsequences have positive LLE and passed the surrogate test.

As presented in Fig. 4, the values of *D*_*q*_, as different fractal dimensions for various *q* measures, as well as those of *f*(*α*), confirm different fractal dimensions. This reveals multi-fractality of the CGR picture. Therefore, the statistical self-similarity of the CGR should be described by a spectrum of fractal dimensions. Figure 5 shows the existence of multi-fractal property demonstrating that the subsequences originated from a deterministic process. It should be noted that multi-fractal curves reveal structural information which is hidden in the original subsequences.

Altogether, these findings indicate that the establishment of full length haplotype sequences can yield new features under the laws of stochastic processes. In addition, these results show that it is possible to extract some other features of subsequences for the purpose of evaluating their similarity and clustering state in a given haplotype by employing chaos theory assumptions. In particular, chaotic analysis reveals the deterministic nature of haplotype sequences. In some problems, such as haplotype assembly, in which the amount of noise and coverage rate of input fragments can affect the quality of reconstructed haplotypes, it is possible to rectify the achieved haplotypes via some features, such as the correlation between neighboring SNPs.

### Exploiting CGR for haplotype reconstruction

**Figure 6:**
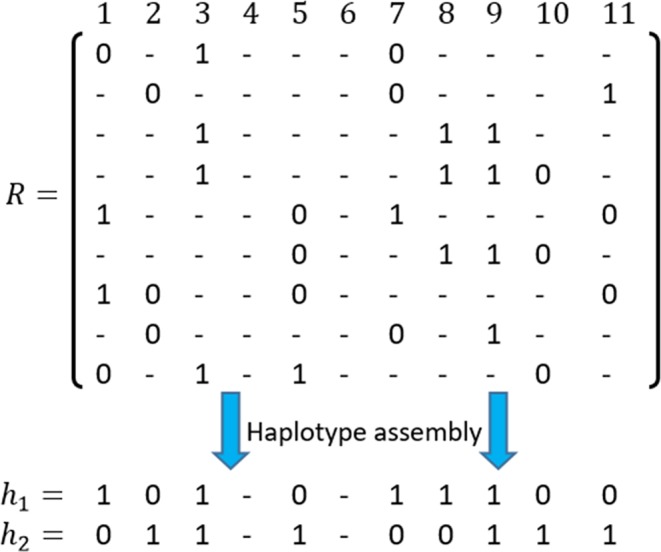
Example of an SIH problem. Matrix *R* contains input fragments. *h*_1_ and *h*_2_ are the reconstructed haplotypes. Positions 4 and 6 are ambiguous because they are not covered by any input fragments.

To improve the reconstruction rate in the single individual haplotype (SIH) problem, one can explore how to use the chaotic feature in the extracted subsequences. In diploid organisms, such as humans, chromosomes are in pairs inherited from the father and mother respectively. As seen in Fig. 6, SIH involves several input fragments, known as *reads* (*r*_*i*_), which are attained from a defined region on a pair chromosomes, based on a sequencing read technology. A set of reads can be represented by matrix *M* × *N*, namely *R*, where each element *r*_*ij*_ belongs to {0, 1, ‘−’}. It is worth to noting that coverage refers to the number of reads that cover a certain position. For example, as can be seen in Fig. 6, coverage of the first column equals with 4, because it has been covered by *r*_1_, *r*_5_, *r*_7_, and *r*_9_. The haplotype assembly attempts to reconstruct haplotypes *h*_1_ and *h*_2_, such that these sequences are the most compatible with the input fragments. As mentioned earlier, the existing approaches show a low performance when matrix *R* involves columns with insufficient coverage. In these columns, there is not sufficient data to determine the measure of alleles with high confidence. Moreover, perhaps there are some positions which are not covered by any input fragments. In such cases, these positions will remain ambiguous and be represented by gaps.

The results in previous sections show the route of chaos in several extracted haplotype subsequences. These findings reveal a dependency among SNPs, which can serve as promising knowledge for determining the measure of alleles in ambiguous positions. For this purpose, it is necessary to first provide test sequences with gap positions. These sequences were obtained by corrupting the evaluated subsequences in a substitution of 10% of the individual subsequences for gaps. Next, each corrupted subsequence was mapped by CGR and its corresponding coordinate series was extracted as mentioned before. When dealing with gap measures, the algorithm was restarted and the next point was added between the center of line and the obtained allele. In the next step, the LP method estimated the allele measures in the gap positions. In deterministic chaotic flows, LP is generally applied for noise reduction. Since chaotic attractors are limited in their phase space, each divergence can be interpreted as noise. Regarding to this fact, the phase space is reconstructed. Afterwards, LP enhances the trajectories of the attractor locally. In particular, for each point, a set of its neighbors is found and a local curve which approximates them is determined. Finally, the considered point is updated by projecting to the resultant curve. Readers interested in the details of the LP algorithm may refer to an excellent book by Kantz and Schreiber^55^.

In this problem, projection was only limited to the points which indicated gap positions. It should be noted that these points were initially set by the average of their neighbors’ measures. Next, a set of neighbors containing 2N + 1 points was constructed, which consisted of the considered point as well as *N* points before and *N* points after the considered point.

**Figure 7:**
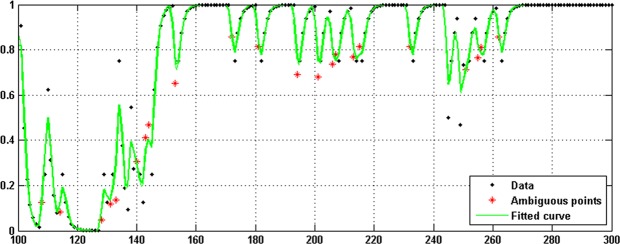
A part of the extracted coordinate series from Chromosome 1’s subsequence (Data), the fitted curve which is the output of the proposed method (Green Curve), and the star points indicating positions with ambiguous values which are determined by a projection to the fitted curve.

Figure 7 demonstrates a part of the coordinate series extracted from the first subsequence of Chromosome 1, which is fitted by the LP method. The star signs indicate positions with ambiguous measures projected to the fitted curve. To specify the allele measure of a gap position in the haplotype, the next step converts the value of the projected point to 0 or 1 by comparisons with the threshold.

14 $$h(i)=\{\begin{array}{ll}0 & if\,cs(i)\le 0.5,\\ 1 & Otherwise,\end{array}$$

where *cs*(*i*) refers to the ith ambiguous measure projected to the fitted curve and *h*(*i*) is the output for the ith position with a gap measure.

**Figure 8:**
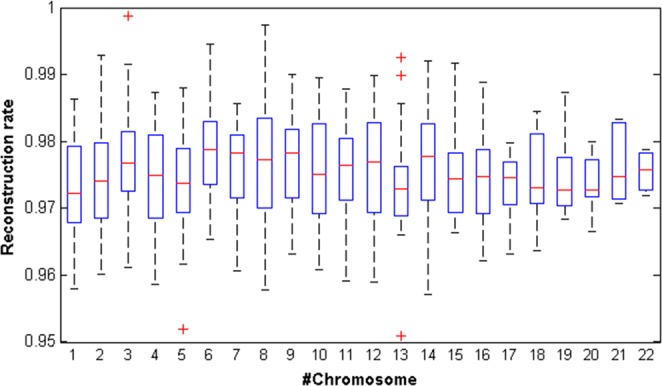
The deviation of reconstruction rates for all extracted subsequences from all chromosomes.

The proposed method was employed for all obtained coordinate series from all subsequences of all chromosomes. The reconstructed subsequences were compared with the original subsequences. Figure 8 depicts the percentage of improvements. As seen in Fig. 8, the boxplot demonstrates the deviation of the reconstruction rate for all subsequences belonging to a specified chromosome. The results demonstrate that the rate of reconstruction for all subsequences was more than 97% overall.

## Conclusion

The current study investigated the chaotic behavior of haplotype sequences by considering the distribution of SNPs and mapping them with the CGR algorithm. The application of surrogate test and multi-fractal analysis procedure on a haplotype dataset demonstrated that the full length of chromosomes did not exhibit chaotic behavior. However, it was found that various numbers of subsequences throughout all haplotypes showed a deterministic nature. According to these findings, the laws of chaotic and stochastic processes can be employed for modeling haplotype sequences in a size-dependent manner. Moreover, the present study applied this knowledge to improve the reconstruction rate in the haplotype assembly problem. These promising results demonstrate that chaotic viewpoint can be effectively utilized to determine alleles in gap positions or low coverage positions. Finally, the source code used in the current work is available from the author upon request.

## Data Availability

The datasets generated and analyzed during the current study are available from the corresponding author on reasonable request.
